# Occlusal Indicators Used in Dental Practice: A Survey Study

**DOI:** 10.1155/2021/2177385

**Published:** 2021-11-03

**Authors:** Tanya Bozhkova, Nina Musurlieva, Diyan Slavchev, Mariana Dimitrova, Sevda Rimalovska

**Affiliations:** ^1^Department of Prosthetic Dentistry, Faculty of Dental medicine, Medical University–Plovdiv, Bulgaria; ^2^Department of Social Medicine and Public Health, Faculty of Public Health, Medical University–Plovdiv, Bulgaria; ^3^Department of Pediatric Dentistry, Faculty of Public Health, Medical University–Plovdiv, Bulgaria

## Abstract

**Introduction:**

The function of the masticatory apparatus is complete when the dentition is intact with contact between the individual teeth and proper occlusion with the antagonists. For years, occlusal contacts have been studied to determine their exact location and describing various materials and methods for their registration such as paper foil, silk, and Shimstock foil. For years, occlusal contacts have been studied to determine their exact location and describe various materials and methods for their registration such as paper foil, silk, shim stock foil, the T-Scan system, and more recently the OccluSense system. The primary aim of the study was at evaluating which of the occlusal indicators is the most commonly used in practice, and the secondary aim was whether dentists are willing to use digital methods to examine occlusion.

**Materials and Methods:**

The main primary information of the survey was collected by sending electronically anonymous questionnaires to 2014 dentists, randomly selected from all regions of the country. 228 questionnaires were filled in and returned. To achieve the goal of the study, the self-developed questionnaire was created and tested to survey the opinion about the use of occlusal indicators in dental practice. Each questionnaire contains questions about the sociodemographic and professional status of the people in the group and their opinion about the positives and negatives and the effectiveness of occlusal indicators.

**Results:**

The obtained results confirm the statement that the most frequently used occlusal indicator in dental practice is the articulation paper. Articulation foil and silk are used less frequently than articulation paper. Of the listed quality indicators, Shimstock foil is rarely used in practice. Of the indicated quantitative indicators, the T-Scan system is more used than the OccluSense system. In the era of rapid technology development, the opinion and desire of dentists to increasingly want to introduce in their clinical practice quantitative methods are the digital diagnosis of occlusion.

**Conclusion:**

In any dental practice, if technically possible, digital methods would be used, giving more accurate and reliable data on the registered occlusal contacts.

## 1. Introduction

The function of the masticatory apparatus is complete when the dentition is intact with contact between the individual teeth and proper occlusion with the antagonists [1]. In this condition, the dentition is a single functional system and the masticatory apparatus is in functionality equilibrium. The size, shape, and arrangement of the teeth are significant for occlusion [2].

With occlusal stability, multiple contacts are created between the teeth, the strongest contacts being on the last pair of the antagonist's teeth [3]. Adequate assessment and control of occlusion are essential for achieving the proper functioning of the masticatory apparatus. Irregular occlusal contacts can lead to congestion of the teeth and the appearance of various clinical symptoms, such as migration of the teeth, cracked enamel, darkening of the teeth, atrophy of periodontal tissue, gingival recession, defects in filling or crowns, osteoarthritis of implants, diseases of the TMJ, migraine, and orofacial pain [4–12].

For years, occlusal contacts have been studied to determine their exact location and describing various materials and methods for their registration such as paper foil, silk, and Shimstock foil. For years, occlusal contacts have been studied to determine their exact location and describe various materials and methods for their registration such as paper foil, silk, shim stock foil, the T-Scan system, and more recently the OccluSense system [13–16]. The occlusal indicators locate and specify the contacts. The accuracy of the applied technique depends on the following factors: thickness, tensile strength, and elasticity of the recording material, the oral environment, and the interpretation of the dentist [17, 18]. The methods for registration of occlusal-articulation relations are qualitative and quantitative [13, 19, 20].

The most commonly used for registration of occlusal contact points are quality indicators, due to their lower price and ease of use. With these materials, it is possible to determine only the location and number of occlusal contacts. The sequence, time, and strength of occlusal contacts can be determined by quantitative methods for the registration of occlusal articulation relations [21, 22].

The primary aim of the study was at evaluating which of the occlusal indicators is the most commonly used in practice, and the secondary aim was whether dentists are willing to use digital methods to examine occlusion.

## 2. Materials and Methods

The main primary information of the survey was collected by sending electronically anonymous questionnaires to 2014 dentists, randomly selected from all regions of the country. 228 questionnaires were filled in and returned (the response rate −14.87%). The minimum sample size of participants was established based on power analysis for sample size calculation. The survey was conducted using the latest Microsoft data processing systems and programs. An operating system was used for this purpose: Microsoft Windows 10 pro; database: Cloud MySQL server; and web server: Windows Server 2019 Datacenter. The server program for collecting and processing the results is Microsoft SharePoint Server 2016.

To achieve the goal of the study, the self-developed questionnaire was created and tested to survey the opinion about the use of occlusal indicators in dental practice. Each questionnaire contains questions about the sociodemographic and professional status of the people in the group and their opinion about the positives and negatives and the effectiveness of occlusal indicators. The ethical committee at MU–Plovdiv approved the questionnaires and the methodology of the survey with its decision no. 4 on 16.10.2014.

The collected primary statistical information was entered and processed with the statistical package SPSS Statistics 25.0, and the graphs were prepared using Microsoft Office 2007. Descriptive statistics for quantitatively measurable quantities and nonparametric tests were used to test hypotheses. For a significance level at which the null hypothesis was rejected, *p* < 0.05 was chosen.

## 3. Results

**Figure 1:**
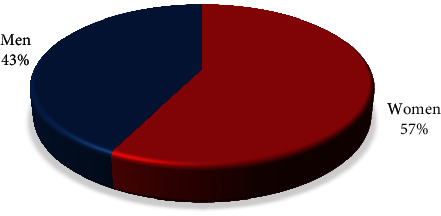
Distribution of respondents by gender.

The study involved 228 patients distributed into 3 age groups (Table 1). The mean age of the participants was 50.35% ± 9.85%. Of the respondents who participated in the online survey, 131 were women (57.46% ± 3.27%) and 97 were men (42.54% ± 3.27%) (Figure 1).

The largest is the relative part of participants in the age range 40–59 years, with 50 (51.55% ± 5.07%) women and 71 (54.20% ± 4.35%) men (Table 1) and with work experience 6–15 years to 78 (34.21% ± 3.14%) (Table 2).

There was no statistically significant difference between the age groups in men and females *p* > 0.05 (*χ*^2^ = 0.67).

**Figure 2:**
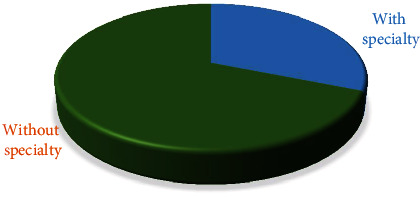
Distribution of dentists according to the availability of the specialty.

Of the 228 dentists participating in the survey, 89 (31.11% ± 2.74%) have acquired a specialty and 139 (68.88% ± 2.74%) have none (Figure 2). From the conducted nonparametric and comparative analyses, no statistically significant differences were found in the structure of the studied sample in terms of postdiploma qualification (*χ*^2^ = 0.55; *p* = 0.814).

**Figure 3:**
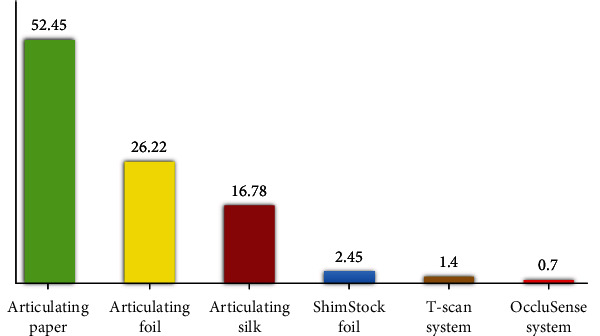
Оcclusal indicators used in practice.

The answers of the dentists to the question “Which occlusal indicators do you use in your practice?” are shown in Figure 3.

**Figure 4:**
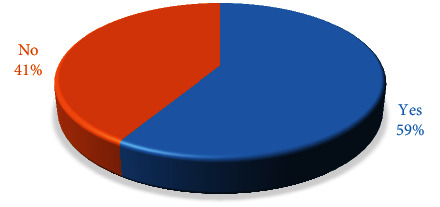
Quantitative occlusal indicator practice.

The answers to the question “Would you use quantitative occlusal indicators in your practice?” are shown in Figure 4.

The chi-square test to check the existing relationship between the opinion of the respondents and the presence of a specialty showed that there was a statistically significant difference in the structure of the answers (*χ*^2^ = 14.73; df = 4; *p* = 0.005).

## 4. Discussion

The result of the analysis of the sociodemographic data of the respondents shows compliance with the global trends for feminization of the profession (women 57.46% ± 3.27%). The reasons can be explained by the possibility of better distribution of working time and the opportunity to develop their practice; on the other hand, this is a profession that requires a sense of empathy and patience, typical of women. The results of many studies show that there are certain models for those involved in surveys. In general, women would be more likely to participate than men, as well as younger people. Similar parameters concerning gender and age were also observed in our sample.

In the literature, many different attempts have been made to classify occlusal indicators. Most of them are divided into two groups—qualitative and quantitative indicators [13, 19, 20].

- The first group includes the articulation paper, articulation foil, articulation, silk, occlusal registers with various imprints, and waxes
- The second group includes the T-Scan system and OccluSense system

The most commonly used of them is the articulation paper [23]. Many types differ in thickness (200 *μ*, 100 *μ*, 60 *μ*, and 40 *μ*), width, material, and type of impregnated dye.

According to some researchers, articulation with silk is the best means of registering occlusal contacts [13, 17]. According to some authors, the interpretation of the markings obtained with the articulation paper is inaccurate, as the assessment of occlusal contacts is subjective and the strength and time of their occurrence can not be determined [24–27]. The quality occlusal indicator disadvantage is that they cannot determine the sequence and strength of contacts, although there are literature data that determine the strength of the contact, according to the intensity of staining. According to other authors, the intensity of the marking is an inaccurate criterion for assessing the strength of occlusal contacts [24, 28].

The fibrous structure determines its high color capacity, and the obtained markings are extremely accurate [13], as almost no false contacts are registered. Articulation foil is the thinnest occlusal indicator and, according to Sharma et al., more accurately registers occlusal contacts between the teeth than the paper and silk [13]. With the help of the Shimstock foil, it is possible to determine whether there is contact between the antagonist teeth [18]. The T-Scan system is a reliable method for recording occlusion, as it quantifies occlusal forces and contact time [29].

The obtained results confirm the statement that the most frequently used occlusal indicator in dental practice is the articulation paper (52.45 ± 2.95%). Articulation foil (26.22 ± 2.6%) and silk (16.78 ± 2.21%) are used less frequently than articulation paper. Of the listed quality indicators, Shimstock foil is rarely used in practice (2.45 ± 0.91%). In our opinion, this is because it does not visualize occlusal contacts on the tooth surface but only determines whether there is contact between the antagonist teeth (18). Of the indicated quantitative indicators, the T-Scan system (1.40 ± 0.69%) is more used than the OccluSense system (0.70 ± 0.49%). In our opinion, the great variety of occlusal indicators is not fully known by dentists. They are not aware of the advantages and disadvantages of different methods.

The T-Scan system has been known to dentists for 34 years, while the OccluSense system has only been on the market for 2 years. Some authors believe that T-Scan technology can be used at any stage of dental treatment related to the diagnosis and correction of occlusion and is one of the practical methods for quantitative analysis of occlusion [25, 30]. T-Scan III achieves even higher pressure distribution during implant treatment [31].

In 2016, Afrashtehfar and Qadeer claimed that the T-Scan system was the preferred clinical tool in the diagnosis of occlusion because it accurately and quickly recorded the distribution of contacts [25].

The dentist receives information that allows it to perform accurate occlusal adjustment when using the T-Scan system [32, 33].

The OccluSense system is an entirely new system for occlusion examination, which was produced in 2019. As it is a relatively new method, there is no literature on its accuracy and reliability in occlusion examination [14].

There is a lot of evidence in the literature for the reliability and help of a T-Scan system in the study of occlusion [15, 16, 25, 32, 33]. The OccluSense system is a new product for which there are no further proven clinical benefits in the clinical study of occlusion [17]. Both systems provide quantitative data on registered occlusal contacts. In the era of rapid technology development, the opinion and desire of dentists to increasingly want to introduce in their clinical practice quantitative methods are the digital diagnosis of occlusion (58.74 ± 2.11%). They provide digital data that facilitates and supports more accurate analysis and more accurate assessment of occlusal contacts. These methods also allow for better communication and motivation of the patient, as his condition can be explained visually. The OccluSense system sensor, thanks to its surface dye, stains the occlusal contacts on the tooth surface [17]. The T-Scan system must always be used in combination with the articulation paper to visualize registered occlusal contacts [33].

## 5. Conclusion

In their clinical practice, dentists most often use articulation paper as an occlusal indicator. There is also a growing interest in quantitative methods for studying occlusion. In any dental practice, if technically possible, digital methods would be used, giving more accurate and reliable data on the registered occlusal contacts.

## Figures and Tables

**Table 1:** Age and gender determination in contingent research.

Age	Men			Women			Total		
	*n*	%	Sp	*n*	%	Sp	*n*	%	Sp
20–39 years	26	26.80	4.50	29	22.14	3.63	55	24.12	2.83
40–59 years	50	51.55	5.07	71	54.20	4.35	121	53.07	3.30
>60 years	21	21.65	4.18	31	23.66	3.71	52	22.81	2.78
Total	97	100.00	—	131	100.00	—	228	100.00	—

**Table 2:** Years of service of respondents.

Years of service of respondents	(*n*)	(%)	Sp
<5 years	61	26.75	2.93
6–15 years	78	34.21	3.14
16–30 years	33	14.47	2.33
>30 years	56	24.56	2.85
Total	228	100.0	—

## Data Availability

Тhe results of the study were collected through an anonymous questionnaire for the study of the methods for registration of occlusal contacts.
